# Supplementary material for: Optimization of differentiation and transcriptomic profile of THP-1 cells into macrophage by PMA
Source: PLoS One. 2023 Jul 17;18(7):e0286056. doi: 10.1371/journal.pone.0286056 (PMC10351730; doi:10.1371/journal.pone.0286056)
Supplement: S1 File — (ZIP) [file pone.0286056.s001.zip › supporting files/RNA-seq report.zip!/RNA-seq report/4_GOEnrichment_result/induce_vs_control/DAG/induce_vs_control.DEG_down_mf_DAG.pdf]

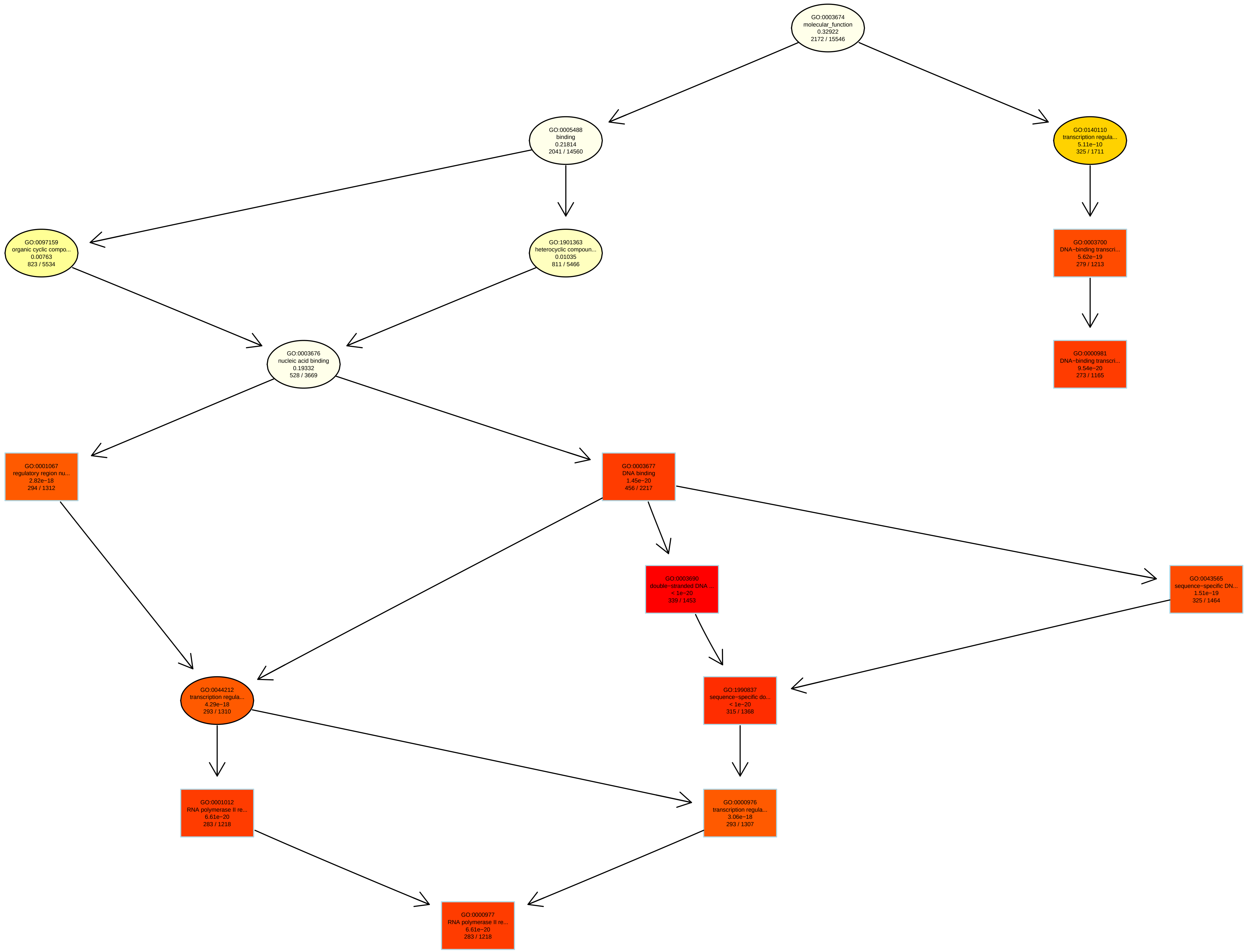
GO:0003674 molecular_function 0.32922 2172 / 15546
GO:0005488 binding 0.21814 2041 / 14560
GO:0140110 transcription regula... 5.11e−10 325 / 1711
GO:0097159 organic cyclic compo... 0.00763 823 / 5534
GO:1901363 heterocyclic compoun... 0.01035 811 / 5466
GO:0003700 DNA−binding transcri... 5.62e−19 279 / 1213
GO:0003676 nucleic acid binding 0.19332 528 / 3669
GO:0000981 DNA−binding transcri... 9.54e−20 273 / 1165
GO:0001067 regulatory region nu... 2.82e−18 294 / 1312
GO:0003677 DNA binding 1.45e−20 456 / 2217
GO:0003690 double−stranded DNA ... < 1e−20 339 / 1453
GO:0043565 sequence−specific DN... 1.51e−19 325 / 1464
GO:0044212 transcription regula... 4.29e−18 293 / 1310
GO:1990837 sequence−specific do... < 1e−20 315 / 1368
GO:0001012 RNA polymerase II re... 6.61e−20 283 / 1218
GO:0000976 transcription regula... 3.06e−18 293 / 1307
GO:0000977 RNA polymerase II re... 6.61e−20 283 / 1218
